# Developing a community-informed sexual and gender minority health research training program in the Deep South

**DOI:** 10.3389/fpubh.2025.1573811

**Published:** 2025-06-26

**Authors:** Emma Sophia Kay, Gabe H. Miller, Frank Puga, Josh Bruce, Bridge Hill Kennedy, Gregory M. Pavela, Erin Densley, Trevis Smith, Mallie Froehlich, Sarah MacCarthy

**Affiliations:** ^1^School of Nursing, University of Alabama at Birmingham, Birmingham, AL, United States; ^2^Department of Sociology, University of Alabama at Birmingham, Birmingham, AL, United States; ^3^School of Nursing, Birmingham AIDS Outreach, Birmingham, AL, United States; ^4^Five Horizons Health Services, Tuscaloosa, AL, United States; ^5^Department of Psychology, University of Alabama at Birmingham, Birmingham, AL, United States; ^6^School of Public Health, University of Alabama at Birmingham, Birmingham, AL, United States; ^7^AIDS Alabama, Birmingham, AL, United States

**Keywords:** sexual and gender minorities, LGBTQ, curriculum, training, education

## Abstract

The largest sexual (e.g., lesbian, gay and bisexual) and gender (e.g., transgender, nonbinary, gender diverse) minority (SGM) population in the United States resides in the Deep South; however, this area has no legal protections for SGM individuals, who experience substantial health inequities. Researchers from the Deep South are consistently overlooked in national dialogs on SGM health, with few SGM health training programs located in this area of the country. In response to these health and sociopolitical disparities and the dearth of regional SGM health training programs, we developed GenderS (Education on Gender and Sex), an innovative research education program led by a community-academic partnership that provides experiential and didactic training in SGM health in the Deep South via an online asynchronous curriculum; a one-week in-person residency in Birmingham, Alabama; and monthly virtual networking salons. In this paper, we describe the theoretical underpinnings of GenderS, the process for developing the curriculum and other program components, our evaluation plan, and lessons learned to date. Through dissemination of a national toolkit that includes templates and examples from GenderS, we can help other organizations adapt our program to their local contexts to train the next generation of SGM health researchers. Although we met challenges in developing recruitment materials for the program due to the enactment of an anti-diversity, equity, and inclusion law in Alabama, enthusiasm for our program remained high, illustrated by receipt of applications from 20 applicants across the United States and Africa.

## Introduction: background and rationale

There are pervasive differences across almost all health outcomes between sexual and gender minority (SGM) individuals compared to their cisgender heterosexual counterparts, with Black and Brown SGM individuals experiencing even greater inequities. The most recent estimates from the U.S. Transgender Survey reported 20% of Black transgender and gender non-conforming people reported living with HIV, compared to about 3% for transgender and gender-diverse people of all races, 2% for the overall Black population, and 0.06% for the overall U.S. population (1). However, these health inequities are not limited to HIV. For example, a recent study found that lesbian and bisexual women had 1.58 and 2.07 times greater odds, respectively, of experiencing multiple conditions and health behaviors, including psychological distress, cigarette smoking and alcohol consumption, compared to their heterosexual counterparts (2).

Although the Deep South (Alabama, Georgia, Louisiana, Mississippi, and South Carolina) is home to the country’s largest SGM population (3), it provides no legal protection for SGM individuals across housing, employment, education, or healthcare sectors. The resulting stigma and discrimination further exacerbate existing health inequities in SGM communities. For example, the Deep South has higher HIV incidence and HIV-related mortality rates than any other area of the country (4, 5), and criminalizes acts that engender potential HIV exposure, without considering the role of prophylactic measures such as condoms and pre-exposure prophylaxis. Further, many of these laws expand beyond healthcare access to include other social determinants of health.

Illustrating this point, in 2024 Alabama passed legislation that prohibits state agencies, public schools, and universities from maintaining or funding diversity, equity, and inclusion (DEI) programs. Additionally, it required public universities to “designate restrooms on the basis of biological sex,” which Alabama law defines as “the physical condition of being male or female, as stated on the individual’s original birth certificate” and not the gender that aligns with how a person identifies. The law, which identified race, sex, gender identity, ethnicity, national origin, and sexual orientation as “Divisive Concepts,” is expected to have a chilling effect on SGM people in the state, especially those at the intersection of other marginalized identities (e.g., race and ethnicity, low socioeconomic status). To underscore the extent of this geographic disparity, a report from the Movement Advancement Project, which monitors U.S. state policies that impact SGM people, illustrates that 93% of SGM people in the South live in negative or low equality states, compared to 0% of SGM people in the Northeast (6).

Although health inequities are especially dramatic in the Deep South, few programs in the region focus on training individuals about how sex and gender affect SGM health. In fact, only one of 25 experts from the South was included in recent national reports on measuring sex, gender identity, and sexual orientation. To address the dearth of SGM-focused training in the Deep South, we developed GenderS (Education on Gender and Sex), an innovative research education program funded by the National Institutes of Health (R25LM014336) and led by a community-academic partnership that draws deeply on our personal and professional experience and expertise navigating SGM health in the Deep South. This paper describes the program’s theoretical underpinnings, the process for developing the curriculum and other program components, our evaluation plan, and lessons learned to date.

## Theoretical underpinnings

### Pedagogical principles

A primary guiding principle of our program is a *community-informed approach*, recognizing that expertise in SGM health is found in a range of experiences and backgrounds beyond academia. Drawing on these diverse sets of skills and experiences is particularly critical in the challenging policy environment of the Deep South, where SGM-serving organizations are accustomed to navigating complex legal and policy environments. To ensure the centering of community voices in our program, our program leadership structure includes both University of Alabama at Birmingham (UAB) faculty and regional community partners (i.e., individuals working at one of five participating community organizations). We intentionally considered the lived experiences of program leadership; thus we have representation from individuals who are SGM (including transgender, queer, and nonbinary), Black, living with HIV, raised in rural parts of the South, and from a military background. At the time of proposal submission, one of the Multiple Principal Investigators (MPIs), ESK, was a researcher at a community organization, further illustrating the joint commitment to an equitable community-academic partnership. This principle is also exemplified in our program’s eligibility criteria: it is open to anyone with a bachelor’s degree in any subject and an interest in SGM health. We established a bachelor’s degree as the minimum requirement to ensure all scholars possess foundational experience in higher education while maintaining accessibility and avoiding overly restrictive criteria. A second principle of the program is a focus on *effecting structural level change* through training the next generation of researchers in both didactic and experiential SGM health content. In the GenderS curriculum, scholars will become familiar with core topics related to SGM health as well as research methods and considerations for addressing SGM health inequities. Through networking salons, scholars will gain the networking skills needed to engage in personal and professional conversations around emerging issues in SGM health. Finally, the in-person residency will offer a nuanced understanding of the systems and structures that perpetuate health inequities in this region, as well as the people and communities who rise above them. Each of these programmatic components are committed to illustrating the risk and resilience of our SGM communities in the Deep South and will support a cohort of future researchers poised to positively impact the health of SGM communities.

### Pedagogical framework

We used the framework of human-centered design (HCD) (7–9) to collaboratively create all aspects of the program, including the curriculum, in-person residency, and networking salons. HCD builds on several principles that are well-suited to program development and integration of diverse voices, such as iterative development and involvement of multi-disciplinary teams (9, 10). This HCD approach fundamentally supports the program’s core mission of equitable partnership across community and academic leaders in the Deep South. By applying the co-creation principles of HCD across each component of the GenderS program, we incorporate community expertise in each aspect of the training program, further amplifying the voices of diverse populations with lived and/or programmatic experience. To illustrate how HCD can be used to design educational content, we provide an overview of how HCD informed the curriculum.

**Figure 1:**
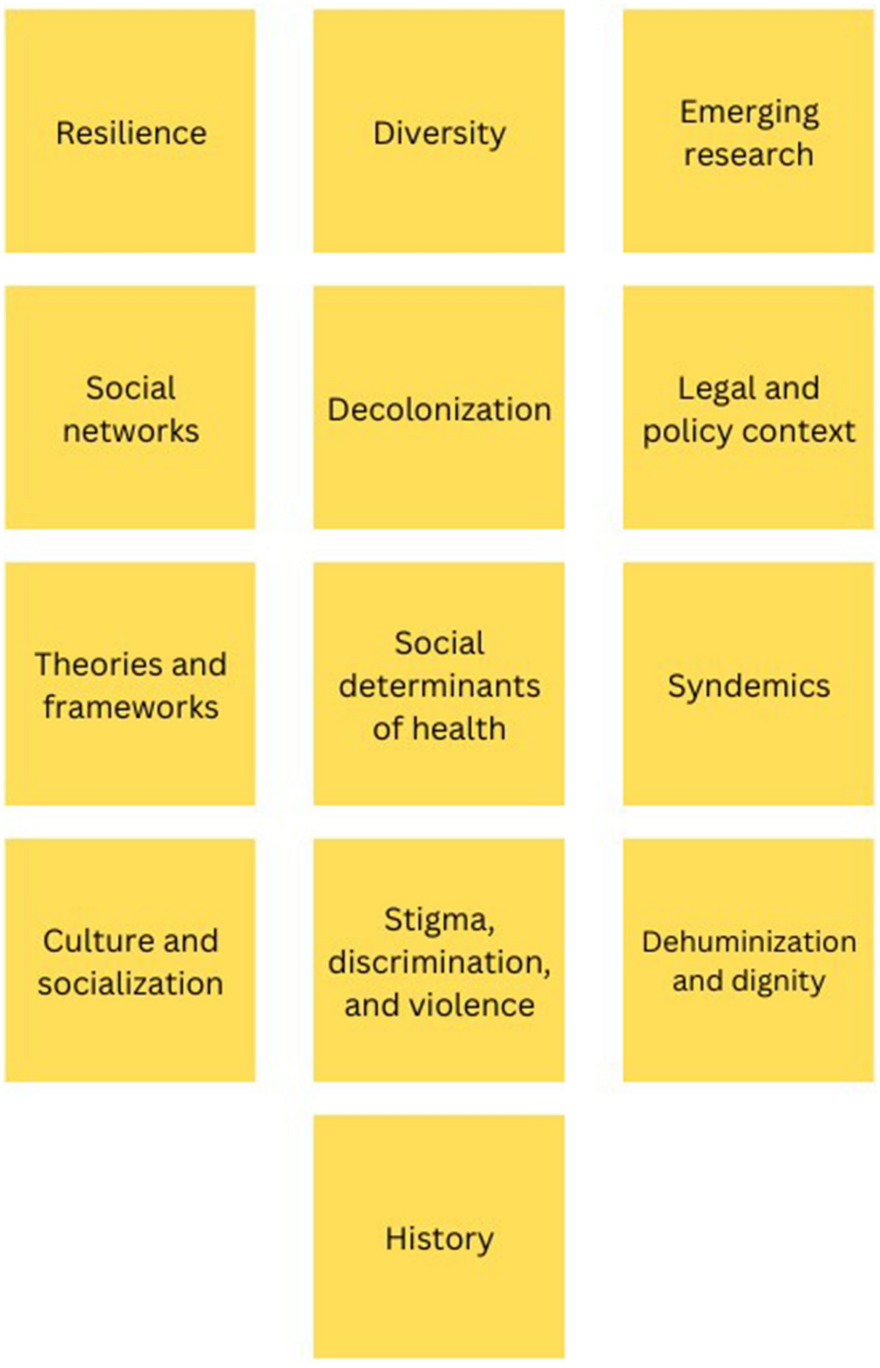
Themes

We used two standard HCD activities to collaboratively identify the major components of our SGM curriculum with our community and academic partners: the *Rose-Thorn-Bud* exercise and the *Affinity Clustering* activity (7). To map out core themes, we used the *Rose-Thorn-Bud* method to highlight aspects of SGM resilience (Rose), disparities experienced by SGM communities (Thorn), and areas where we can improve SGM health using the resources available in the Deep South (Bud). Everyone was provided colored Post-it notes on which to write Roses (pink), Thorns (blue), and Buds (green), with one idea per note. Then, we used *Affinity Clustering* to group these factors into common themes. Participants were encouraged to place their Post-it notes on a whiteboard, one at a time, while reading their idea aloud to the group. Over the course of the activity, participants began to identify emerging themes among groups of notes, which were physically clustered according to similarity. Figure 1 illustrates the final list of curricular themes prioritized in the HCD session.

Using this final list, we developed a broad course outline for a 14-week curriculum to further discuss and refine with faculty and community partners (see Supplementary material). During a one-hour meeting with faculty and community partners, we finalized the curriculum through iterative discussion, including adding a presentation on disability and including a separate module for race/ethnicity. This same framework continues to underpin our design and implementation of the training program and includes the in-person residency and networking salons, providing an ongoing feedback loop for all partners to contribute and improve the program in real time.

## Learning environment

### Program overview

**Figure 2:**
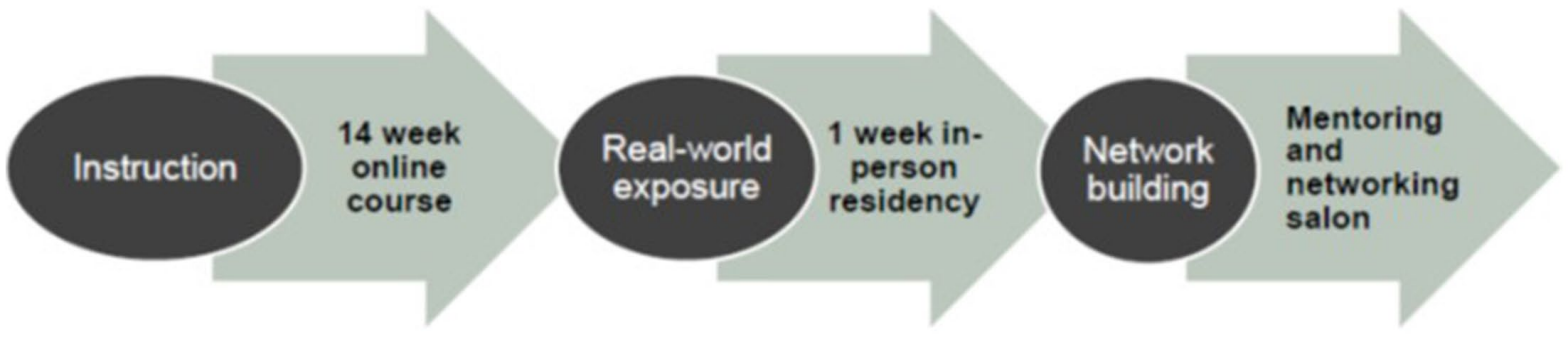
Core components of the GenderS program.

GenderS is a 1-year educational program that includes both online and in-person learning experiences. The program offers a variety of learning and networking opportunities for scholars to increase and apply knowledge in SGM health, thereby accommodating a range of learning styles and methods. The three primary components include an asynchronous online curriculum; a one-week in-person residency in Birmingham, Alabama; and monthly virtual mentoring and networking salons (Figure 2). Scholars also have monthly meetings with their program mentors, who include our community and faculty partners. Two cohorts of 7–10 scholars each will complete the one-year program in Years 2 and 3, with Year 1 dedicated to program development and Year 4 to evaluation and dissemination.

### Scholar recruitment and selection

The launch of recruitment efforts coincided with enactment of Alabama’s Senate Bill 129 (11), one component of which prevents universities from sponsoring DEI programs. This required a re-imagining of how to meaningfully advance core components of the program. In collaboration with our program’s leadership team, and with constant review and feedback from institutional leadership across the UAB campus, we developed recruitment materials and launched a virtual campaign to raise program visibility. We identified and met with a university-approved marketing firm that specializes in SGM marketing and advertising. The firm created an ad campaign targeting eligible potential applicants based on education (e.g., having at minimum a bachelor’s degree) and career interests (e.g., SGM health, public health, health policy). The ad campaign, which ran for 1 month on LinkedIn in the early fall of 2024, received 58,600 impressions (i.e., how many times it was seen by potential applicants). Additionally, the MPIs, co-investigators, and academic and community partners shared the flyer with their personal and professional networks.

Applications were collectively reviewed and scored by the leadership team using an NIH scoring system (i.e., 1 to 9) across three domains: clear articulation of personal and professional goals, alignment of these goals with SGM health in the Deep South, and personal and professional experiences. Finalists were interviewed via Zoom by the MPIs. We received 20 applications for the first cohort, of whom 10 were selected. Five have a bachelor’s degree, three have a masters, and two have a doctorate. The applicants are also geographically diverse. Over half (60%) are from the South, with 50% from the Deep South; one is from the Midwest (10%); two are from the Northeast (20%); and one is from Africa (10%).

### Learning objectives

Learning objectives for GenderS map onto four fundamental learning areas that are realized across the three programmatic components. These learning areas include core knowledge and skills (curriculum), networking and professional relationships (mentoring and networking salons, in-person residency), practical experience and community engagement (mentoring and networking salons, in-person residency), and research and professional development (mentoring and networking salons, curriculum, and in-person residency). By the end of the program, scholars should be able to:

1. Describe health risk behaviors and outcomes that disproportionately impact SGM people in general and especially in the Deep South.
2. Develop professional networks within the SGM health field.
3. Connect the concepts discussed in the online course with how those concepts look “on the ground.”
4. Engage in research and advocacy related to SGM health.

### Asynchronous online curriculum

#### Format and content

The asynchronous online curriculum is divided into 14 modules, which can be completed online and asynchronously by program scholars. Each module is led by one or more members of the core program team and includes between three and six content areas (apart from the final module, which provides a conclusion to the course). Broadly speaking, the course covers core concepts in sex/gender, SGM theories (e.g., intersectionality, minority stress, structural stigma), SGM health (e.g., mental and behavioral health, HIV, substance use), SGM health and social institutions (e.g., media and culture, military) research methods, and policy. Individual content areas constitute a 5–20-min video that is developed and led by an MPI, co-investigator, faculty partner, or community partner who has expertise in that area. Each recording is also accompanied by suggested readings selected by the video lead, as well as “listening checks” that assess scholars’ mastery of the content. All videos were professionally recorded in a studio on UAB’s campus.

We also incorporated short interviews into the asynchronous online curriculum—“conversations” with faculty and community partners to demonstrate a wide range of experiences and career paths. Each ~10-min conversation explores the individual’s career path, including reasons for being interested in SGM health, as well as reflections on what they would tell their “younger self.” Academic partner conversations demonstrate various pathways to conducting SGM research in university settings, while community partner conversations highlight the ways in which SGM expertise is found in a range of settings. These partner conversations also demonstrate to program scholars that there is no singular “right” career path, and that oftentimes career trajectories are nonlinear and even unexpected.

#### E-learning platform

The online course for both cohorts is hosted on the university’s professional studies Canvas eLearning platform. We felt the professional studies Canvas platform traditionally used for continuing education students was appropriate, as most scholars were not affiliated with UAB. Before developing the course, the MPIs met with the eLearning team to discuss the cost of hosting the course and the general requirements for recording each section. The eLearning team recorded and uploaded all content videos except academic and community partner videos, which were recorded via Zoom. Two members of the eLearning team created individual modules corresponding with the curriculum in Canvas and uploaded all syllabus readings. Before the online course’s launch, the eLearning team sent individual registration links to give program participants access to the platform for the entirety of the training program.

### In-person residency

The GenderS program features a one-week residency in Birmingham, Alabama, designed to increase knowledge related to SGM health experientially by connecting the concepts covered in the asynchronous course with how those concepts are operationalized in community settings. Each day will include a combination of site visits to different community partner organizations highlighted below.

- At AIDS Alabama, scholars will learn how the organization addresses the needs of Latine and Black persons living with and at increased risk of HIV acquisition through its medical services, mental health services, vocational training services, and bilingual case management.
- At Birmingham AIDS Outreach (BAO), scholars will tour multiple affiliated facilities including the Magic City Wellness Center (the only SGM primary care facility in Alabama), Magic City Acceptance Academy (the region’s first SGM charter school), and Magic City Acceptance Center (a resource center for SGM youth). BAO is on the frontlines of serving SGM people in Birmingham, AL across the lifespan, despite originating as the state’s first AIDS Service Organization.
- At the Birmingham Civil Rights Institute, scholars will tour this internationally recognized site and learn about the intersections of the civil rights and SGM rights movements.
- Five Horizons Health Services is the largest HIV provider in Alabama, serving patients across some of the most rural areas of Alabama and into Mississippi. Scholars will learn how the organization meets the health needs of its rural catchment area across five pillars: medical care, prevention and testing, supportive services, advocacy, and research.
- At the Hispanic and Immigrant Center of Alabama (¡HICA!), scholars will learn how the organization champions economic equality, civic engagement, and social justice for Latine families in Alabama, and, increasingly, the SGM community.

The in-person residency will conclude with Birmingham’s annual pride parade, which gives scholars the opportunity to celebrate the strength and resiliency of the SGM community with each other, faculty partners, and community partners.

### Networking salons and mentor matching

The GenderS program’s networking salons, modeled after Dr. Lisa Bowleg’s Intersectionality Training Institute (12), create an informal space for exchanging research ideas, thoughts, and opinions in a relaxed yet intellectually stimulating environment. These monthly virtual salons will give scholars the opportunity to connect with others informally and create a safe space to talk about how SGM health is framed in the media, via two primary formats:

- “Ripped from the Headlines” conversations center discussion on a recent news story, examining how it is relevant to SGM health.
- “Viewing Parties” involve discussion of how SGM health is portrayed in TV and film.

Each salon is facilitated by the MPIs and one or more faculty or community partners. These discussions provide opportunities for scholars to discuss media influences on the portrayal of SGM health, building on knowledge gained in the curriculum (e.g., the module on Media and Culture). The salon format also facilitates networking among scholars, faculty partners, and community partners.

Scholars also receive tailored mentoring from a faculty or community partner. Mentors are matched according to scholars’ preferences and provide monthly one-on-one meetings structured to achieve scholars’ personal and professional goals and objectives. They may also meet face-to-face with scholars during the in-person residency. The program’s mentoring component offers flexible guidelines that accommodate both the diverse interests and needs of scholars and the varied expertise of mentors. Mentors can tailor their approach based on the needs of the scholars. Some focus on providing targeted feedback on dissertations and research papers, while others share guidance in career navigation and professional development within the SGM health field.

## Assessment

### Program evaluation plan

Each GenderS scholar completes a 5-min online survey upon program enrollment that encompasses the four primary learning areas mentioned previously: (1) Core knowledge and skills; (2) Networking and professional relationships; (3) Practical experience and community engagement; (4) Research and professional development. Within 30 days of the conclusion of the program, all scholars will be asked to complete a post-program survey covering the same SGM topics; the results of this pre/post survey will be used to evaluate scholars’ gains in knowledge. The survey will also collect demographic information to monitor the aggregate number and demographic characteristics of participants, including their educational level and degree program, and will ask how they learned of the program to guide future recruitment efforts. In addition, scholars will complete a post-program interview to further contextualize the survey data. Once a year, program mentors, community partners, and faculty partners will also complete an evaluation interview to assess their feedback on (1) program activities, (2) the scholar(s) they mentored, if applicable, and (3) suggestions for program improvement. External evaluators will complete the evaluation and provide the program team with an evaluation report for each cohort of scholars to facilitate continuous programmatic improvement.

## Discussion of practical implications, constraints, and lessons learned

We believe creation of GenderS has important practical consequences. To our knowledge, it is the first SGM health-focused R25 program in the Deep South and, as such, represents an important step toward increasing SGM health knowledge among future researchers, ultimately improving SGM health outcomes. Moreover, through contextualizing the curriculum to the Deep South, we promote knowledge-building in the context of the most conservative area of the country. The program will prepare scholars to address SGM health disparities in any challenging political and legal context: scholars trained in the Deep South will be exceptionally well prepared to navigate structural barriers.

Notably, the constraints faced when launching the program were significant. The anti-DEI bill (SB 129) was going into effect at the same time we were recruiting and launching the application. As a result, we navigated complex conversations regarding what language we could use to talk about GenderS, what questions we could ask applicants, and how to ensure we could achieve our stated goals. Yet, the implementation of the law did not dampen enthusiasm for our program—we received 20 applications, with 60% of applicants residing in the South, 25% of applicants residing in the Northeast or Mid-Atlantic, 5% of applicants residing in the Midwest, and 10% of applicants residing abroad in Africa. Moreover, all the applicant interviews occurred immediately after the 2024 presidential election, and many applicants noted that the coming change in administration further fueled their desire to participate in a training program explicitly focused on how to advance SGM health in a complex political environment. Several applicants also reflected on how the shift in the national legal and policy context raised their interest in learning from researchers and community members in the Deep South about how to effectively navigate SGM health research in a challenging political climate.

These constraints also necessitated significant innovation and lessons learned. First, our community-academic partnership created a broad network of support and expertise in navigating the state anti-DEI law. Our community partners—many of whom have been advocating for SGM rights for decades—were instrumental in identifying how we could effectively “do the work” despite political complexities. Second, we learned how to successfully advocate for collection of broader applicant demographic data (e.g., race/ethnicity and disability) by citing federal data collection guidelines and NIH reporting requirements, which ultimately supported the collection of these important data on program scholars.

To disseminate this program and our lessons learned, we will share key components of our GenderS program in a toolkit that can be tailored to the local context of other areas beyond the Deep South. Our toolkit will include the asynchronous online curriculum, residency example and template, mentoring and networking example and template, and evaluation example and template that organizations and institutions can use to build their own SGM research training program. Ultimately, the toolkit will enable others to learn from our experience and discover how to adapt our program to their own cultural and geographic contexts. National dissemination of the toolkit will also help raise visibility of the community-academic expertise located in the Deep South.

Going forward, we anticipate this program will serve as a testament to the incredible risk and resilience of those doing SGM-focused work in the Deep South and provide guidance on how to advance this research across varied policy environments. Given the growing anti-SGM sentiment in the US, the successes of Deep South community organizations and academic institutions in navigating anti-DEI policies may prove particularly instructive for regions that have historically had stronger legislative protections.

## Data Availability

The original contributions presented in the study are included in the article/Supplementary material, further inquiries can be directed to the corresponding author.
