# Supplementary material for: Developing a community-informed sexual and gender minority health research training program in the Deep South
Source: Front Public Health. 2025 Jun 26;13:1573811. doi: 10.3389/fpubh.2025.1573811 (PMC12243932; doi:10.3389/fpubh.2025.1573811)
Supplement: Supplementary file 1 [file Image_1.pdf]

CENTER FOR THE STUDY OF SEXUAL AND GENDER HEALTH
The University of Alabama at Birmingham

GenderS Training Program
Online Course Curriculum Plan

Module 1 - Introduction to Course

1. Welcome to GenderS Training Program
2. Introduction to the Course
3. Community Partner Conversation
4. Community Partner Conversation
5. Community Partner Conversation
6. Academic Partner Conversation
7. Academic Partner Conversation
8. Academic Partner Conversation

Module 2 - Defining Core Concepts

1. Sex & Gender
2. Sexual Orientation & Gender Identity
3. Measurement of LGBTQ+ Populations
4. History
5. Community Partner Conversation

Module 3 - LGBTQ+ Theories

1. SGMRO LGBTQ+ Health Disparities Framework
2. Minority Stress; Gender Minority Stress Resilience
3. Structural Stigma
4. Life Course
5. Intersectionality

Module 4 - LGBTQ+ Health and Intersectionality

1. Race/Ethnicity
2. Ability Status
3. LGBTQ+ Health Across the Life Course
4. Rurality & Regionality
5. SOGI Subgroup Differences
6. Academic Partner Conversation
7. Community Partner Conversation

Module 5 - LGBTQ+ Health I

1. Exploring Health Behaviors in LGBTQ Populations
2. Examining Health Behaviors in LGBTQ Populations
3. Chronic Illness & Conditions (including cancer)
4. Academic Partner Conversation

Module 6 - LGBTQ+ Health II

1. Reproductive & Sexual Health
2. HIV/AIDS
3. Community Partner Conversation
4. Community Partner Conversation
5. Community Partner Conversation
6. Academic Partner Conversations

Module 7 - LGBTQ+ Health III

1. Mental Health
2. Suicide & Self-Injury
3. Disordered Eating
4. Community Partner Conversation
5. Gender Dysphoria & Gender Affirming Care
6. Community Partner Conversation

Module 8 - Trauma & Violence

1. Trauma (historical/intergenerational)
2. Individual Violence
3. Structural Violence

Module 9 - Conducting LGBTQ+ Research

1. Community Engaged Research, CBPR
2. Qualitative & Quantitative Considerations
3. Academic Partner Conversation
4. Academic Partner Conversation

Module 10 - LGBTQ+ Health & Social Institutions

1. Media and Culture
2. Family & Social Networks
3. SES/Education/Class
4. Military
5. Academic Partner Conversation

Module 11 - Race/Ethnicity and LGBTQ+ Populations

1. NIH SGMRO Symposium Keynote
2. Community Partner Conversation
3. Latinx LGBTQ+ Populations
4. Community Partner Conversation
5. Academic Partner Conversation

Module 12 - Political & Policy Determinants of Health

1. Political & Policy Determinants
2. Community Partner Conversation
3. Community Partner Conversation
4. Community Partner Conversation

Module 13 -LGBTQ+ Advocacy - Law, Policy, and Programs

1. Community Partner Conversation
2. Community Partner Conversation
3. Human Rights

Module 14 - LGBTQ+ Advocacy - Leaders in the Field s Bringing it all Together

1. Bringing it all Together
